# Optimal Conspicuity of Liver Metastases in Virtual Monochromatic Imaging Reconstructions on a Novel Photon-Counting Detector CT—Effect of keV Settings and BMI

**DOI:** 10.3390/diagnostics12051231

**Published:** 2022-05-14

**Authors:** Stefanie Bette, Josua A. Decker, Franziska M. Braun, Judith Becker, Mark Haerting, Thomas Haeckel, Michael Gebhard, Franka Risch, Piotr Woźnicki, Christian Scheurig-Muenkler, Thomas J. Kroencke, Florian Schwarz

**Affiliations:** 1 Clinic for Diagnostic and Interventional Radiology and Neuroradiology, University Hospital Augsburg, Stenglinstr. 2, 86156 Augsburg, Germany; stefanie.bette@uk-augsburg.de (S.B.); josua.decker@uk-augsburg.de (J.A.D.); franziska.braun@uk-augsburg.de (F.M.B.); judith.becker@uk-augsburg.de (J.B.); mark.haerting@uk-augsburg.de (M.H.); thomas.haeckel@uk-augsburg.de (T.H.); michael.gebhard@uk-augsburg.de (M.G.); franka.risch@uk-augsburg.de (F.R.); piotrekwoznicki@gmail.com (P.W.); christian.scheurig@uk-augsburg.de (C.S.-M.); florian.schwarz@uk-augsburg.de (F.S.); 2 Medical Faculty, Ludwig Maximilian University of Munich, Geschwister-Scholl-Platz 1, 80539 Munich, Germany

**Keywords:** photon-counting detector CT, virtual monoenergetic imaging, liver metastases, oncologic imaging

## Abstract

In dual-energy CT datasets, the conspicuity of liver metastases can be enhanced by virtual monoenergetic imaging (VMI) reconstructions at low keV levels. Our study investigated whether this effect can be reproduced in photon-counting detector CT (PCD-CT) datasets. We analyzed 100 patients with liver metastases who had undergone contrast-enhanced CT of the abdomen on a PCD-CT (*n* = 50) or energy-integrating detector CT (EID-CT, single-energy mode, *n* = 50). PCD-VMI-reconstructions were performed at various keV levels. Identical regions of interest were positioned in metastases, normal liver, and other defined locations assessing image noise, tumor-to-liver ratio (TLR), and contrast-to-noise ratio (CNR). Patients were compared inter-individually. Subgroup analyses were performed according to BMI. On the PCD-CT, noise and CNR peaked at the low end of the keV spectrum. In comparison with the EID-CT, PCD-VMI-reconstructions exhibited lower image noise (at 70 keV) but higher CNR (for ≤70 keV), despite similar CTDIs. Comparing high- and low-BMI patients, CTDI-upregulation was more modest for the PCD-CT but still resulted in similar noise levels and preserved CNR, unlike the EID-CT. In conclusion, PCD-CT VMIs in oncologic patients demonstrated reduced image noise–compared to a standard EID-CT–and improved conspicuity of hypovascularized liver metastases at low keV values. Patients with higher BMIs especially benefited from constant image noise and preservation of lesion conspicuity, despite a more moderate upregulation of CTDI.

## 1. Introduction

Contrast-enhanced CT of the abdomen is the most frequently performed test to detect abdominal metastases in patients with known or suspected malignancies [1]. Over recent years, several studies have pointed out the benefits of virtual monoenergetic imaging (VMI) reconstructions derived from venous phase dual-energy CT (DECT) datasets for detecting hypovascularized liver metastases [2,3,4,5,6,7,8,9,10]. As X-ray attenuation of iodine disproportionally increases at lower X-ray energies, the iodine signal is enhanced, improving contrast between metastases and normal liver parenchyma. Conspicuity of liver metastases thus increases at lower kiloelectronvolt (keV) levels [2,4]. However, this gain in iodine signal is accompanied by substantial increases in image noise in lower keV VMI reconstructions from dual-energy datasets [2,11,12].

Contemporary clinical CT scanners are equipped with energy-integrating detectors (EID), converting X-ray photons into light which secondarily creates an electric signal. On the other hand, Photon-Counting Detector CT (PCD-CT) systems implement a novel X-ray detection mechanism: X-ray photons are directly converted in a semiconductor crystal (cadmium-telluride), preserving photon energy information [13,14,15]. Benefits of this technology include electronic noise reduction, improved spatial resolution, and intrinsic spectral sensitivity [13,14,15,16], providing spectral information for every scan. With the broader availability of PCD-CT technology, the routine acquisition of spectral information should substantially enhance its somewhat hesitant clinical adoption.

Being a novel technology, knowledge about VMIs derived from PCD-CT datasets (PCD-VMI) is limited. Recent studies have investigated the value of VMIs for e.g. CT angiography of the aorta, contrast-enhanced abdominal CT, musculoskeletal imaging and emphysema quantification [17,18,19,20]. To our knowledge, there are no studies regarding the potentially improved conspicuity of hypovascularized liver metastases.

Our study aimed to identify the optimal VMI range for this application by analyzing image noise, tumor-to-liver ratio (TLR), and contrast-to-noise ratio (CNR) in various VMIs derived from PCD-CT datasets for the detection of hypovascularized liver metastases.

## 2. Materials and Methods

The local institutional review board approved this retrospective single-center study, and the need to obtain informed consent was waived. The local database was queried for patients with known or suspected malignancies who had undergone contrast-enhanced CT of the abdomen (±chest) on a novel Photon-Counting CT Scanner (NAEOTOM Alpha, Siemens Healthineers, Erlangen, Germany) as part of routine clinical care between April 2021 and July 2021. Accessing electronic medical records, all patients with known liver metastases at the time of scan were included in this analysis.

As a control group, consecutive oncologic patients with known liver metastases who had undergone contrast-enhanced abdominal CT on an EID-CT (20-slice MDCT Somatom AS20, Siemens Healthineers) between October 2020 and July 2021 were also identified and analyzed. 

### 2.1. Scan Protocol and Reconstruction Settings

PCD-CT scans were performed on a photon-counting detector CT (NAEOTOM Alpha, Siemens Healthineers) as routine clinical acquisitions using a biphasic contrast injection protocol. A contrast bolus of 120 mL (iopromide; Ultravist 300 mgI/mL, Bayer, Leverkusen, Germany) injected via an antecubital vein was followed by a 30 mL saline bolus (flow rate: 4.0 mL/s). The scan was bolus-triggered with a delay of 45 s after an attenuation of 120 HU was reached in the ascending aorta. Patients were scanned craniocaudally in a supine position from the diaphragm or upper thoracic aperture to the symphysis during a single breath-hold. The following parameters were applied: an acquisition mode with readout of spectral information (QuantumPlus, Siemens Healthineers, with the following detector-based primary energy thresholds: 20, 35, 65, and 70 keV), 120 kVp tube voltage, automatic tube current modulation (Care DOSE 4D, Siemens Healthineers) with an Image Quality Level of 145; 0.25 s rot. time, 0.8 pitch, 144 × 0.4 mm collimation. Spectral series were generated using a soft-tissue kernel specifically developed for the spectral postprocessing of PCD-CT datasets (Qr40, QIR 3, Siemens Healthineers) and an enhanced DICOM file format containing spectral information (SPP, spectral postprocessing). Slice thickness was 1.0 mm with an increment of 1.0 mm.

In the comparison group, all patients were scanned on an EID-CT (Somatom AS20, Siemens Healthineers) in a supine position using a craniocaudal scan direction from the diaphragm or upper thoracic aperture to the symphysis during a single breath-hold and using an identical contrast material protocol. The following parameters were applied: single-energy mode, CareDose 4D and AutokV (with mAs_ref_ = 165 mAs for 100 kVp and 120 mAs for 120 kVp), 0.5 s rot. time, pitch 1.05, 1.5 × 16 × 1.2 mm collimation. Reconstructions were performed using a soft-tissue kernel (I31f) with a slice thickness of 1.5 mm and an increment of 1.0 mm and raw-data-based iterative reconstruction (SAFIRE, level 3).

### 2.2. Image Postprocessing and Analysis

Postprocessing of PCD-CT data and polychromatic EID-CT data was performed on a dedicated workstation (Syngo.via VB60A, Siemens Healthineers). Using SPP-series, PCD-VMI reconstructions with a slice thickness of 1 mm, increment of 1 mm, identical Field-of-View (FoV) and Z-axis alignment were generated at the following keV levels: 40 keV, 45 keV, 50 keV, 55 keV, 60 keV, 70 keV, 80 keV, 90 keV, 100 keV, 110 keV, 130 keV, 150 keV, 170 keV, 190 keV. EID-CT data were reformatted to 1.0 mm slice thickness and 1.0 mm increment accordingly.

**Figure 1:**
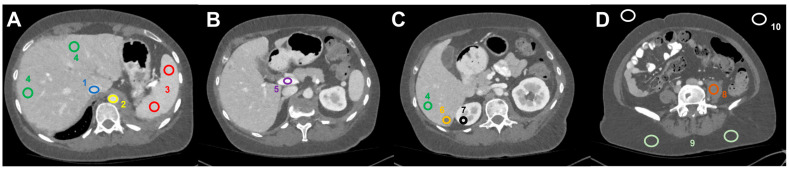
Image analysis and ROI-based measurement of mean HU values (and standard deviation) in dedicated regions: (**A**) (1) inferior vena cava, (2) abdominal aorta, (3) spleen, (4) liver parenchyma, (**B**) (5) portal vein, (**C**) (6) liver metastasis, (7) right renal cortex, (**D**) (8) left psoas muscle, (9) subcutaneous tissue (2×), and (10) air (2×).

Image analysis was performed using the open-source software Fiji [21], an image processing package based on Image J. For each patient, 18 Regions of Interest (ROI)–with a size individually selected as large as possible while still encompassing homogenous tissue areas–were manually positioned in the following distinct anatomic regions on the 70 keV dataset and automatically copied to all other VMI-datasets (Figure 1): liver metastasis (3 ROIs per keV level); normal liver parenchyma (3 ROIs per keV level); inferior vena cava; aorta; portal vein; spleen (2 ROIs); renal cortex (right side); psoas muscle (left side); subcutaneous fat (right and left side); air (3 ROIs). ROIs were positioned in lesions with the highest visual conspicuity in patients with more than three metastases. Positioning of ROIs in EID-CT series was performed in analogy.

A radiologist with eight years of CT experience (S.B.) positioned all ROIs. Mean and standard deviation of CT values (in HU) were calculated from all ROIs. Summary raw data from all measured ROIs are shown in Table S1. Median noise was calculated as the median of SDs in subcutaneous fat.

Tumor-to-liver contrast was calculated as the ratio between CT values measured in metastases and normal liver parenchyma. Ratios were calculated as the mean of all three ROIs per region, resulting in a single tumor-to-liver (TLR) ratio per keV level. Contrast-to-noise ratio (CNR) was calculated as follows:

$$\mathrm{CNR}=\frac{{\mathrm{CTvalues}}_{\mathrm{liver}}-{\;\mathrm{CTvalues}}_{\mathrm{metastases}}}{{\mathrm{SD}\;\mathrm{CT}\;\mathrm{values}}_{\mathrm{subcutaneous}\;\mathrm{fat}}}$$

### 2.3. Weight- and BMI-Dependent Analyses

For BMI- and weight-dependent analyses of image quality and radiation dose, weight and height were derived from individual electronic medical records. Patients were excluded only from weight-dependent analyses if they suffered from severe obesity (BMI > 40 kg/m^2^), if data about BMI or dose were missing, or if patients were unable to raise their arms over their heads during the scan. Median split analysis was performed to compare patients in the high-BMI vs. low-BMI groups [22]. For this analysis patients from the EID-CT group and the PCD-CT group were pooled.

### 2.4. Statistical Analysis

Analysis of descriptive data and statistical analyses were performed using SPSS 28.0 (SPSS Inc., IBM Corp., Armonk, NY, USA). Most variables (CTDI, BMI, noise, CNR, and tumor-to-liver ratio) did not follow a normal distribution and were reported as median and interquartile range (IQR) unless otherwise stated. Mann-Whitney-U tests were performed to compare differences between groups. For intra-individual comparisons, the Wilcoxon signed-rank test for paired samples was used after dichotomization of variables using a 70 keV threshold. Correlations were performed using the Spearman-Rho correlation coefficient. Statistically significant differences were assumed for *p*-values < 0.05. Bonferroni correction was performed to correct for multiple testing [23].

## 3. Results

### 3.1. Patient Population

Overall, 477 oncologic patients were screened (consecutive within scanner-subgroups, PCD-CT: 230; EID-CT: 247) who had undergone contrast-enhanced CT of the abdomen (±chest), and 122 patients (PCD-CT: 60; EID-CT: 62) with known liver metastases were identified. Of these, 22 patients were excluded (PCD-CT: 10, EID-CT: 12, resulting in final cohort sizes of PCD-CT: 50 and EID-CT: 50) due to the following reasons: metastases were hypervascularized (*n* = 3), patients were post local therapy of metastases (e.g., transarterial chemoembolization; *n* = 4), lesions exhibited very low lesion conspicuity (*n* = 12) or small size (<10 mm, *n* = 3) due to response to systemic therapy. In the end, 100 patients with liver metastases were analyzed for this study (57 men; age 65.1 ± 11.9 years [range: 32–88]), 50 of whom had been scanned on the PCD-CT (PCD-CT cohort) and 50 on the EID-CT (EID-CT cohort).

Both cohorts had undergone CT as part of routine clinical care; the PCD-CT cohort between April 2021 and July 2021, the EID-CT cohort between October 2020 and March 2021. On the EID-CT system, 100 kV tube voltage was applied in 46 patients, 120 kV tube voltage in 4 patients. Across all patients, body mass index (BMI) was 23.9 kg/m^2^ [20.54; 26.24]. The most common underlying malignancies were colorectal cancer (*n* = 30), pancreatic cancer (*n* = 19), and lung cancer (*n* = 8). There were no significant differences between groups regarding demographic and baseline clinical data (Table 1).

### 3.2. Image Noise

**Figure 2:**
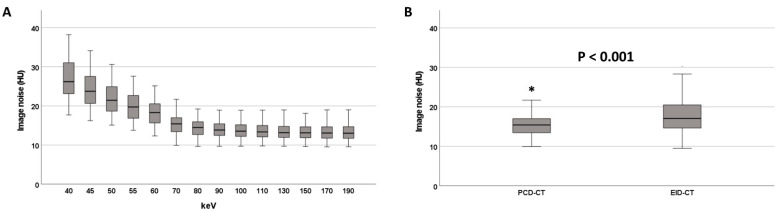
Image noise at different keV levels on the PCD-CT (**A**). Image noise on the PCD-CT (70 keV) and the EID-CT (**B**). * denotes an outlier value.

In the PCD-CT group, image noise substantially decreased between 40 keV and 100 keV VMI (from 26.2 HU [23.1; 31.0] to 13.5 HU [12.2; 15.3]) but did not improve further at higher keV levels (Table 2, Figure 2). Image noise was higher at ≤70 keV compared to >70 keV (20.4 HU [17.0; 24.2] vs. 13.4 HU [12.1; 15.2], *p* < 0.001). 70 keV VMI reconstructions exhibited significantly lower image noise than EID-CT reconstructions (15.4 HU [13.3; 17.0] vs. 17.1 HU [14.6; 20.6], *p* < 0.001, Figure 2, Table S2).

### 3.3. Tumor-to-Liver Ratio (TLR)

**Figure 3:**
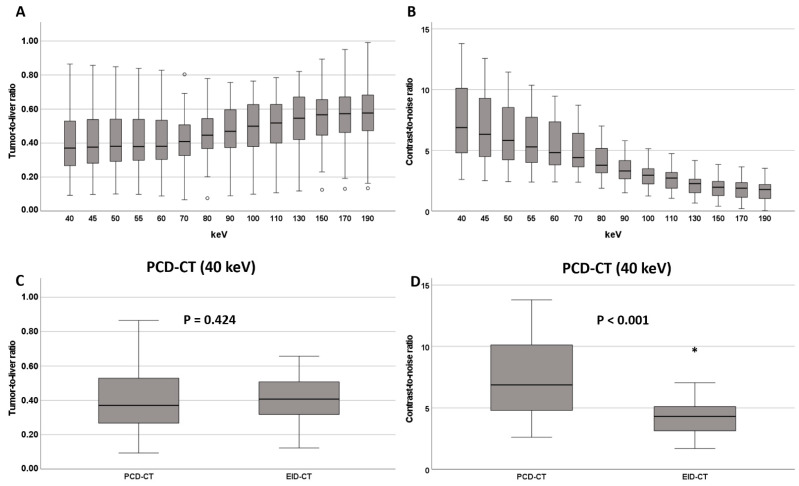
(**A**) Tumor-to-liver ratio and (**B**) contrast-to-noise ratio at different keV levels on the PCD-CT. Comparison of tumor-to-liver ratio and CNR between EID-CT and PCD-CT at 40 keV (**C**,**D**). * and ◦ denote outliers.

In the PCD-CT group, TLR was lowest (and thus conspicuity highest) at the low keV end of the VMI spectrum (40 keV: 0.37 [0.27; 0.53], Figure 3, Table 2). With increasing keV levels, TLR increased steadily, reaching 0.58 [0.47; 0.69] at 190 keV VMI reconstructions. TLRs for VMIs ≤ 70 keV (0.38 [0.29; 0.54]) were significantly lower than for VMIs > 70 keV (0.53 [0.41; 0.63], *p* < 0.001). Reconstructions at 70 keV VMI (PCD-CT) had similar TLRs to those of EID-CT datasets (0.41 [0.33; 0.52] vs. 0.41 [0.32; 0.51], *p* = 0.756, Table 2), while lower keV VMI reconstructions showed significantly lower TLRs and thus higher lesion conspicuity (Figure 3, Table 2).

### 3.4. Contrast-to-Noise Ratio (CNR)

In the PCD-CT group, contrast-to-noise ratio (CNR) had its maximum at the low end of the keV spectrum (6.88 [4.79; 10.19]) and continuously decreased with increasing keV levels, reaching 1.78 [1.02; 2.22] at 190 keV (Figure 3, Table 2). Significantly higher CNRs were observed for VMIs ≤ 70 keV (5.57 [4.12; 7.79]) compared to VMIs > 70 keV (2.44 [1.78; 3.34], *p* < 0.001).

**Figure 4:**
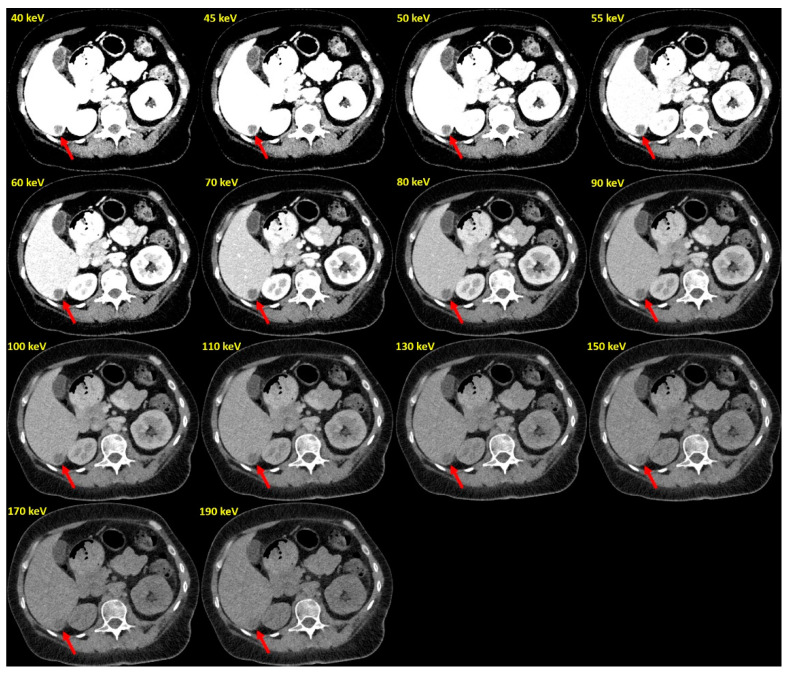
Example of PCD-VMIs (40–190 keV) in a patient with hypovascularized liver metastases of pleura mesothelioma. Hypovascularized metastasis in segment VI (arrow).

Reconstructions at 70 keV VMI (PCD-CT) showed similar CNR to the EID-CT-series (4.41 [3.64; 6.45] vs. 4.31 [3.11; 5.17] *p* = 0.054). At lower keV levels (<70 keV), CNRs of PCD-CT datasets exceeded those of EID-CT datasets (Figure 3 and Figure 4 and Table 2).

### 3.5. Effect of BMI

Ninety-seven patients were included in this analysis. Patients were excluded for the following reasons: BMI > 40 kg/m^2^ (*n* = 1), missing BMI value (*n* = 1) and inability to lift the arm during image acquisition (*n* = 1). Median split for these 97 patients revealed a BMI of 23.9 kg/m^2^ for dichotomization.

For analyses including CTDI-values, due to missing CTDI values for two patients, 95 patients were included. 

Analysis of dose modulation revealed the expected positive correlation between BMI and CTDI for both scanner systems (PCD-CT: rho = 0.76, *p* < 0.001; EID-CT: rho = 0.79, *p* < 0.001), and between weight and CTDI (PCD-CT: rho = 0.73, *p* < 0.001; EID-CT: rho = 0.81; *p* < 0.001). However, the EID-CT system exhibited a more pronounced increase of CTDI at higher BMI values (Figure S1).

**Figure 5:**
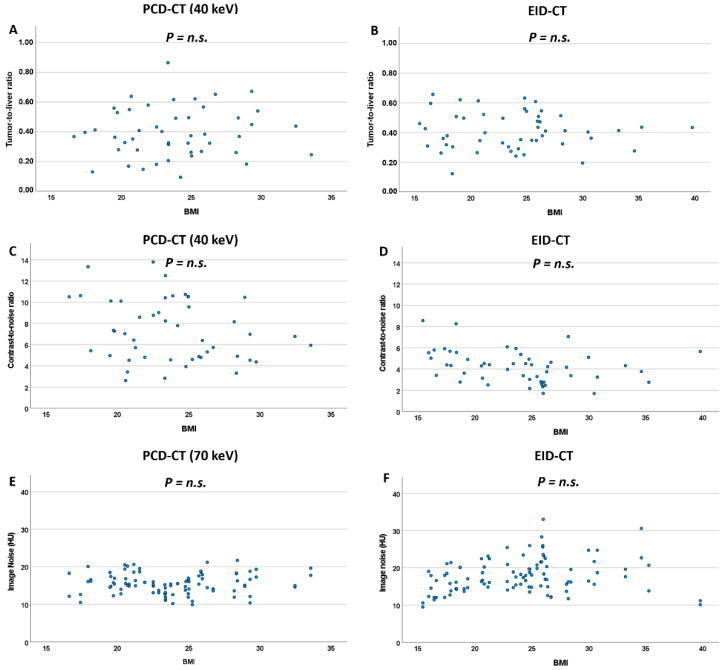
Scatterplots for TLR (**A**,**B**), CNR (**C**,**D**) and image noise (**E**,**F**) against BMI for the PCD-CT and EID-CT system. n.s.: non significant.

Comparing image noise in high-BMI vs. low-BMI patients, high-BMI patients exhibited slightly higher image noise on the EID-CT system (18.3 HU [15.5; 21.9] vs. 16.1 HU [14.2; 18.9], *p* = 0.011, n.s. after Bonferroni correction). On the PCD-CT system, there was no difference in image noise between high-BMI and low-BMI patients for the central 60–80 keV range of VMI reconstructions (for 70 keV: 15.8 HU [14.1; 17.8] vs. 15.3 HU [13.1; 16.8]; *p* = 0.382). For all other keV levels, image noise was higher in high-BMI patients (Table 3). These findings are further supported by analyzing correlations between BMI and image noise at various keV levels (Figure 5, Table S4).

Differences in TLR between high-BMI and low-BMI patients were not significant, neither on the EID-CT system nor on the PCD-CT system (Figure 5, Table 4). Concordantly, correlations between BMI and TLR were not significant, neither for the EID-CT nor any PCD-VMI.

CNR, on the other hand, showed significant differences between high-BMI and low-BMI patients on the EID-CT system (3.37 [2.76; 4.51] vs. 4.52 [3.96; 5.79], *p* = 0.003) but not for the majority of keV levels (40–120 keV) in PCD-VMI reconstructions. Concordantly, there was a correlation between BMI and CNR for the EID-CT system (rho = −0.399, *p* = 0.004), whereas BMI and CNR lacked significant correlation for all keV levels in PCD-VMIs and showed lower rho-values (Figure 5, Table S4).

## 4. Discussion

This study reports our initial experience with VMI reconstructions from PCD-CT datasets in 50 patients with hypovascularized liver metastases, compared to 50 similar patients scanned on an EID-CT. The most important results of our study are as follows: first, maximal conspicuity of liver metastases was observed for PCD-VMIs at the lowest keV levels, which far exceeded that of EID-CT acquisitions using identical contrast protocols and similar radiation doses. Second, PCD-VMIs in the 40–70 keV range consistently exhibited higher CNR and lower image noise at 70 keV than EID-CT acquisitions. Third and most importantly, despite similar BMI-dependent dose modulation, CNR was preserved across a broad BMI spectrum on the PCD-CT system while exhibiting a highly negative correlation with BMI on the EID-CT system.

This study systematically compared objective image quality parameters of VMI reconstructions derived from PCD-CT datasets in 50 patients with hypovascularized liver metastases and 50 similar patients scanned on a modern EID-CT. CT values in metastases, normal liver, and other defined abdominal locations were measured for a wide range of PCD-VMIs (40–190 keV) and on regular reconstructions for EID-CT datasets. It is well known that for EID-DECT acquisitions, lower keV levels in VMI reconstructions improve the visualization of hypovascularized liver metastases [2,4,24]. The present study extends these findings to PCD-CT systems: at lower keV levels, the observed changes in TLR and CNR expressed substantially improved lesion conspicuity. Maximal CNR was observed at 40 keV and far exceeded that of the EID-CT. Our results are in line with a recent study on abdominal PCD-CT, in which Higashigaito et al. reported higher CNRs at similar subjective image quality at 50 keV compared to EID-CT [20].

Previous studies using EID-DECT reported higher image noise at lower keV levels [2,11]. Concordantly, we observed higher image noise at PCD-VMI levels of 40–70 keV. These results agree with a recent study on the image quality of VMI reconstructions derived from contrast-enhanced abdominal PCD-CT [20]. Importantly, however, in the study by Higashigaito et al., subjective image quality at lower keV levels was rated higher despite higher image noise, suggesting that image noise is secondary to CNR in terms of subjective image quality in abdominal CT imaging. On the other hand, at 70 keV (and higher keV levels), PCD-VMI reconstructions exhibited considerably less image noise than EID-CT reconstructions.

In future studies, it will be important to pinpoint exactly the optimal keV setting for specific abdominal pathologies. CNR and TLR showed the highest conspicuity of liver metastases at 40 keV, but at this keV level the image noise was higher. Considering the recent study by Higashigaito et al., image noise might be secondary to CNR and TLR, suggesting lower keV levels for conspicuity of liver metastases [20]. There might also be differences in the optimal keV levels for different pathologies and organs and for different patients (e.g., regarding BMI). For clinical routine, it might be necessary to find the optimal keV value for the best consideration of the whole abdomen. Further prospective studies are necessary to address this issue.

Despite active dose-modulation, higher BMI is usually associated with lower EID-CT image quality due to higher image noise [25]. Our study reports similar findings for PCD-CT datasets: patients with higher BMI-values exhibited significantly higher image noise on both CT systems and at all analyzed PCD-VMI keV levels. There was no correlation between BMI and TLR values, neither for the EID-CT nor any PCD-VMI keV-level. As expected, higher BMI values were associated with lower CNR and thus lower lesion conspicuity on the EID-CT. However, most interestingly, no correlation between BMI and CNR was observed for the PCD-CT system, suggesting that CNR is preserved across a wide range of BMI values. These results are in agreement with a previous study suggesting the benefit of using PCD-CT for overweight patients [19].

One of the significant innovations of the PCD-CT technology is the routine acquisition of spectral information at every scan. When it comes to analyzing hypovascularized liver metastases, additional VMI-reconstructions at low keV levels can help better delineate liver metastases in clinical routine.

This study has several limitations. First, this is a retrospective single-center study based on our initial experience with a novel photon-counting CT. All images were acquired as part of routine clinical care. As described above, there are differences in acquisition parameters between PCD-CT and EID-CT (e.g., reconstruction kernels). To eliminate potential bias that might arise from the differences in slice thickness of primary reconstructions (EID-CT: 1.5 mm, PCD-CT: 1.0 mm, each with a 1.0 mm increment), all data were reformatted to a slice thickness of 1.0 mm with a 1.0 mm increment.

Second, there were differences in tube modulation and tube control: EID-CT used automated tube current and tube voltage selection, whereas PCD-CT used automated tube modulation only. Third, we included patients with liver metastases of different primary tumors, which might have introduced some bias. Forth, only quantitative data about tumor-to-liver ratio, image noise, and contrast-to-noise ratio were analyzed. As these quantitative data suggest a benefit to using VMIs at lower keV levels for detecting hypovascularized LMs, further studies with qualitative analyses and data about subjective image quality are necessary to comprehensively evaluate their diagnostic utility.

## 5. Conclusions

In conclusion, our initial experience with PCD-CT VMIs in oncologic patients demonstrates reduced image noise in comparison with a standard EID-CT, and improved conspicuity of hypovascularized liver metastases at lower keV levels. Our results suggest that patients with higher BMI benefit especially from PCD-CT over EID-CT: despite more moderate upregulation of CTDI, image noise is unchanged and lesion conspicuity well preserved in comparison with low-BMI patients.

## Figures and Tables

**Table 1:** Patient Characteristics.

Parameter	PCD-CT	EID-CT	*p*-Value
**Age**, years	65.0 ± 12.4 (32–85)	65.7 ± 11.8 (32–88)	0.597
**Sex**, male	29/50	28/50	0.693
**BMI**, kg/m^2^	23.3 [20.6; 25.9]	24.4 [19.0; 26.3]	0.934
**CTDI**, mGy	6.7 [5.9; 8.1]	6.4 [4.9; 8.4]	0.128
**DLP,** mGy*cm	402.0 [339.0; 491.0]	378.9 [274.4; 492.9]	0.362
**Primary Malignancy**			
● Colorectal cancer	13	17	
● Pancreatic cancer	11	8	
● Lung cancer	5	3	
● Other	21	22	

Normally distributed data shown as mean ± SD and range, non-normally distributed data shown as median [interquartile range]. PCD: Photon-Counting Detector; EID: Energy-Integrating Detector; BMI: Body-Mass-Index; CTDI: CT Dose Index.

**Table 2:** Tumor-to-liver ratio and contrast-to-noise ratio at different keV levels.

keV Level	Tumor-to-Liver Ratio (TLR)			Contrast-to-Noise Ratio (CNR)		
	PCD-CT	EID-CT	*p*-Value	PCD-CT	EID-CT	*p*-Value
40	0.37 (0.27–0.53)	0.41 (0.32–0.51)	0.42	6.88 (4.79–10.19)	4.31 (3.11–5.17)	*<0.001*
45	0.38 (0.28–0.54)		0.54	6.32 (4.46–9.30)		*<0.001*
50	0.38 (0.29–0.54)		0.67	5.81 (4.22–8.59)		*<0.001*
55	0.38 (0.30–0.54)		0.79	5.28 (3.98–7.76)		*<0.001*
60	0.38 (0.30–0.54)		0.85	4.82 (3.79–7.35)		0.006
70	0.41 (0.33–0.52)		0.76	4.41 (3.64–6.45)		0.054
80	0.45 (0.36–0.55)		0.12	3.78 (3.13–5.23)		0.788
90	0.47 (0.37–0.60)		0.02	3.30 (2.65–4.24)		0.014
100	0.50 (0.38–0.63)		*0.003*	2.96 (2.21–3.56)		*<0.001*
110	0.52 (0.40–0.63)		*<0.001*	2.72 (1.87–3.21)		*<0.001*
130	0.55 (0.42–0.67)		*<0.001*	2.26 (1.51–2.67)		*<0.001*
150	0.57 (0.45–0.66)		*<0.001*	1.96 (1.26–2.48)		*<0.001*
170	0.57 (0.46–0.68)		*<0.001*	1.88 (1.11–2.36)		*<0.001*
190	0.58 (0.47–0.69)		*<0.001*	1.78 (1.02–2.22)		*<0.001*

Data shown as median (interquartile range), *p*-values < 0.0035 shown in *italics*; PCD: Photon-Counting Detector; EID: Energy-Integrating Detector.

**Table 3:** Image noise at different keV levels in BMI-subgroups after median split.

	PCD-CT			EID-CT		
keV	Noise, HU		*p*-Value	Noise, HU		*p*-Value
	*BMI ≤ 23.9 kg/m^2^*	*BMI > 23.9 kg/m^2^*		*BMI ≤ 23.9 kg/m^2^*	*BMI > 23.9 kg/m^2^*	
40	24.2 [22.4; 27.6]	27.9 [24.5; 32.1]	*0.002*	16.1 [14.2; 18.9]	18.3 [15.5; 21.9]	0.011
45	21.8 [20.1; 24.9]	24.8 [21.6; 28.4]	0.006			
50	19.9 [18.2; 22.6]	22.5 [19.6; 25.6]	0.006			
55	18.6 [16.4; 21.0]	20.7 [17.9; 23.2]	*0.025*			
60	17.9 [15.2; 19.4]	19.3 [16.4; 21.6]	0.074			
70	15.3 [13.1; 16.8]	15.8 [14.1; 17.8]	0.382			
80	14.0 [12.3; 15.3]	15.1 [13.4; 16.4]	0.067			
90	13.6 [11.9; 14.7]	14.6 [12.9; 16.1]	0.022			
100	13.2 [11.7; 14.2]	14.5 [12.9; 16.1]	0.009			
110	13.0 [11.6; 14.1]	14.5 [12.9; 16.2]	0.007			
130	12.9 [11.4; 13.9]	14.3 [12.8; 16.1]	0.004			
150	12.7 [11.4; 13.9]	14.3 [12.7; 16.1]	*0.003*			
170	12.6 [11.3; 13.8]	14.3 [12.7; 16.1]	*0.002*			
190	12.5 [11.3; 13.8]	14.3 [12.7; 16.1]	*0.002*			

Median of SD’s measured in subcutaneous fat. Data shown as median [interquartile range], *p*-value < 0.0033 shown in *Italics*. PCD: Photon-Counting Detector; EID: Energy-Integrating Detector; BMI: Body-Mass-Index.

**Table 4:** Tumor-to-liver ratio and contrast-to-noise ratio at different keV levels for PCD-CT and EID-CT, divided in BMI subgroups by median split.

	**PCD-CT**					
**keV**	**Tumor-to-Liver Ratio (TLR)**		***p***	**Contrast-to-Noise Ratio (CNR)**		***p***
	***BMI ≤ 23.9***	***BMI > 23.9***		***BMI ≤ 23.9***	***BMI > 23.9***	
40	0.38 [0.28; 0.53]	0.37 [0.26; 0.52]	0.98	7.79 [4.92; 10.44]	5.93 [4.70; 8.86]	0.22
45	0.38 [0.29; 0.54]	0.38 [0.27; 0.53]	0.98	7.28 [4.56; 9.41]	5.46 [4.40; 7.88]	0.21
50	0.39 [0.30; 0.55]	0.38 [0.29; 0.53]	0.97	6.65 [4.31; 8.61]	5.04 [4.13; 7.88]	0.22
55	0.38 [0.30; 0.55]	0.38 [0.30; 0.54]	0.95	6.08 [4.18; 7.95]	4.62 [3.86; 7.25]	0.18
60	0.38 [0.29; 0.54]	0.38 [0.30; 0.53]	0.80	5.60 [4.11; 7.64]	4.25 [3.64; 7.05]	0.20
70	0.41 [0.30; 0.52]	0.41 [0.33; 0.55]	0.73	4.92 [3.79; 6.81]	3.98 [3.55; 6.33]	0.18
80	0.45 [0.33; 0.54]	0.45 [0.38; 0.58]	0.56	4.02 [3.41; 5.93]	3.27 [2.85; 5.01]	0.11
90	0.45 [0.35; 0.60]	0.49 [0.39; 0.63]	0.55	3.55 [2.83; 5.06]	2.84 [2.32; 4.10]	0.08
100	0.50 [0.38; 0.63]	0.52 [0.40; 0.68]	0.44	3.12 [2.23; 4.48]	2.53 [2.00; 3.44]	0.10
110	0.52 [0.40; 0.63]	0.54 [0.43; 0.71]	0.40	2.84 [2.03; 4.06]	2.30 [1.75; 2.93]	0.06
130	0.54 [0.42; 0.64]	0.56 [0.47; 0.75]	0.35	2.47 [1.83; 3.48]	2.00 [1.33; 2.40]	0.03
150	0.56 [0.44; 0.65]	0.59 [0.50; 0.77]	0.30	2.23 [1.65; 3.05]	1.82 [1.06; 2.10]	0.02
170	0.57 [0.45; 0.64]	0.59 [0.52; 0.79]	0.27	2.08 [1.53; 2.76]	1.63 [0.89; 1.93]	0.02
190	0.57 [0.46; 0.64]	0.60 [0.53; 0.81]	0.27	1.98 [1.45; 2.58]	1.48 [0.77; 1.81]	0.02
	**EID-CT**					
	**Tumor-to-Liver Ratio (TLR)**		***p***	**Contrast-to-Noise Ratio (CNR)**		***p***
	***BMI ≤ 23.9***	***BMI > 23.9***		***BMI ≤ 23.9***	***BMI > 23.9***	
	0.38 [0.30; 0.51]	0.41 [0.35; 0.51]	0.69	4.52 [3.96; 5.79]	3.37 [2.76; 4.51]	*0.003*

Data shown as median (interquartile range), *p*-values < 0.0033 shown in *italics.* PCD: Photon-Counting Detector; EID: Energy-Integrating Detector; BMI: Body-Mass-Index.

## Data Availability

The data presented in this study are available on request from the corresponding author.

## Supplementary Materials

The following supporting information can be downloaded at: https://www.mdpi.com/article/10.3390/diagnostics12051231/s1, Figure S1: Correlations between CTDI and BMI and CTDI and weight for PCD-CT (A,C) and EID-CT (B,D). Table S1: ROI measurements/Summary Raw Data; Table S2: Image noise in PCD-VMIs at different keV levels and in EID-CT datasets; Table S3: Median image noise at different keV levels; Table S4: Spearman correlations between BMI, image noise, TLR and CNR.
