# Supplementary material for: Optimal Conspicuity of Liver Metastases in Virtual Monochromatic Imaging Reconstructions on a Novel Photon-Counting Detector CT—Effect of keV Settings and BMI
Source: Diagnostics (Basel). 2022 May 14;12(5):1231. doi: 10.3390/diagnostics12051231 (PMC9140684; doi:10.3390/diagnostics12051231)
Supplement: Supplementary file 1 [file diagnostics-12-01231-s001.zip › Tabel S2.pdf]

Supplemental Table S2 Median image noise at different keV levels

	PCD-CT	EID-CT	P-Value
keV	Noise (median SD)		
40	30.6 (24.5-36.2)	17.6 (13.8-21.5)	*<0.001*
45	27.2 (21.8-32.2)		*<0.001*
50	24.5 (19.7-28.8)		*<0.001*
55	22.2 (18.0-25.9)		*<0.001*
60	20.2 (16.6-23.7)		*<0.001*
70	16.6 (13.8-19.1)		*<0.001*
80	15.3 (13.0-17.4)		*<0.001*
90	14.7 (12.6-16.8)		*<0.001*
100	14.5 (12.5-16.7)		*<0.001*
110	14.4 (12.4-16.5)		*<0.001*
130	14.4 (12.3-16.5)		*<0.001*
150	14.3 (12.2-16.4)		*<0.001*
170	14.3 (12.2-16.4)		*<0.001*
190	14.3 (12.2-16.4)		*<0.001*

Median of all measured SD's. Data shown as median (interquartile range), P-Value < 0.0035 shown in *italics*
