# Supplementary material for: Optimal Conspicuity of Liver Metastases in Virtual Monochromatic Imaging Reconstructions on a Novel Photon-Counting Detector CT—Effect of keV Settings and BMI
Source: Diagnostics (Basel). 2022 May 14;12(5):1231. doi: 10.3390/diagnostics12051231 (PMC9140684; doi:10.3390/diagnostics12051231)
Supplement: Supplementary file 1 [file diagnostics-12-01231-s001.zip › Table S1.pdf]

Table S1 ROI measurements / Summary Raw Data

PCD-CT			
**keV**	**Location**	**Mean (HU)**	**SD (HU)**
40	Liver metastases	108.8 [72.9; 173.1]	33.9 [29.8; 37.4]
	Liver parenchyma	311.6 [261.1; 360.9]	31.1 [28.3; 36.3]
	VCI	546.2 [456.1; 617.3]	36.9 [30.4; 41.8]
	Aorta	579.1 [518.9; 656.4]	30.7 [26.7; 36.1]
	Portal vein	657.7 [605.9; 740.7]	39.9 [34.9; 48.8]
	Spleen	420.2 [375.7; 489.1]	33.1 [29.4; 37.1]
	Renal cortex	687.9 [609.2; 738.1]	37.5 [33.1; 50.5]
	Muscle	98.6 [86.7; 113.8]	30.2 [27.3; 32.8]
	Subcutaneous	-156.8 [-171.5; -125.8]	26.2 [23.1; 31.0]
	Air	-998.4 [-999.9; -996.4]	17.1 [14.9; 20.1]
45	Liver metastases	92.5 [62.7; 145.4]	30.1 [26.8; 33.1]
	Liver parenchyma	258.1 [220.3; 297.5]	27.8 [25.1; 32.4]
	VCI	443.2 [371.0; 492.9]	32.5 [26.8; 37.0]
	Aorta	464.5 [421.8; 534.7]	27.2 [23.6; 31.2]
	Portal vein	534.9 [496.1; 600.3]	35.4 [31.0; 40.6]
	Spleen	341.9 [309.3; 397.6]	29.4 [26.0; 32.3]
	Renal cortex	549.9 [496.9; 598.6]	32.8 [28.2; 42.9]
	Muscle	87.2 [76.4; 100.3]	26.6 [24.5; 29.1]
	Subcutaneous	-139.6 [-152.4; -113.8]	23.7 [20.6; 27.6]
	Air	-998.6 [-999.7; -997.1]	15.3 [13.4; 17.9]
50	Liver metastases	79.9 [57.3; 123.6]	27.1 [24.3; 29.9]
	Liver parenchyma	219.1 [188.3; 252.4]	25.3 [22.7; 28.9]
	VCI	365.4 [304.1; 414.7]	28.2 [24.5; 33.1]
	Aorta	385.4 [347.3; 440.1]	24.3 [21.5; 27.8]
	Portal vein	437.5 [407.5; 493.9]	31.2 [27.9; 36.1]
	Spleen	286.8 [257.3; 332.9]	26.4 [23.4; 29.1]
	Renal cortex	451.9 [403.3; 491.7]	29.9 [25.2; 37.2]
	Muscle	78.2 [69.9; 89.8]	24.2 [22.1; 26.8]
	Subcutaneous	-127.4 [-138.1; -104.0]	21.5 [18.6; 24.9]
	Air	-998.7 [-999.5; -997.6]	13.8 [12.0; 16.3]
55	Liver metastases	69.6 [50.7; 103.7]	24.3 [22.1; 27.0]
	Liver parenchyma	188.1 [162.6; 213.4]	22.7 [20.4; 26.0]
	VCI	304.3 [253.5; 336.5]	25.4 [21.7; 29.5]
	Aorta	319.1 [288.3; 364.4]	21.8 [19.6; 25.2]
	Portal vein	362.4 [339.5; 412.1]	27.7 [25.2; 32.8]
	Spleen	240.3 [216.7; 275.7]	23.8 [21.4; 26.6]
	Renal cortex	372.5 [334.2; 405.5]	27.1 [22.5; 33.1]
	Muscle	71.4 [63.8; 81.5]	22.1 [19.9; 24.1]
	Subcutaneous	-118.3 [-125.9; -96.4]	19.7 [16.9; 22.7]
	Air	-998.8 [-999.5; -997.8]	12.5 [10.8; 14.7]
60	Liver metastases	61.2 [44.5; 89.5]	22.2 [20.0; 24.5]
	Liver parenchyma	163.7 [142.7; 183.4]	20.8 [18.6; 23.6]
	VCI	257.6 [215.9; 284.8]	22.6 [19.2; 26.9]
	Aorta	267.4 [242.4; 307.8]	20.0 [17.7; 22.9]
	Portal vein	304.6 [286.7; 348.7]	25.4 [22.5; 29.7]
	Spleen	204.7 [185.9; 233.6]	21.7 [19.5; 24.6]
	Renal cortex	311.2 [281.8; 339.9]	25.0 [19.8; 29.4]
	Muscle	66.4 [59.9; 75.3]	20.0 [17.9; 22.6]
	Subcutaneous	-110.2 [-117.2; -90.7]	18.3 [15.7; 20.6]

	Air	-998.7 [-999.4; -998.1]	11.2 [9.7; 13.0]
70	Liver metastases	51.9 [38.4; 70.7]	18.2 [16.3; 20.2]
	Liver parenchyma	127.1 [114.9; 142.5]	17.1 [15.2; 18.8]
	VCI	189.9 [159.7; 206.5]	18.3 [16.1; 20.6]
	Aorta	197.4 [178.3; 227.6]	15.7 [13.9; 18.3]
	Portal vein	222.0 [206.1; 257.9]	20.4 [17.8; 23.5]
	Spleen	155.3 [141.0; 170.1]	17.4 [16.1; 19.6]
	Renal cortex	225.0 [206.4; 247.3]	20.1 [15.9; 22.6]
	Muscle	59.3 [53.4; 66.0]	16.9 [14.4; 18.8]
	Subcutaneous	-99.1 [-105.0; -82.5]	15.4 [13.3; 17.0]
	Air	-998.9 [-999.4; -998.4]	9.3 [7.9; 11.3]
80	Liver metastases	47.3 [35.0; 61.2]	16.7 [14.9; 18.4]
	Liver parenchyma	104.9 [96.4; 116.2]	15.7 [14.2; 17.5]
	VCI	146.2 [125.9; 157.5]	16.1 [14.5; 17.7]
	Aorta	151.9 [138.1; 174.7]	14.5 [13.1; 16.9]
	Portal vein	168.4 [156.3; 197.2]	18.7 [16.2; 21.0]
	Spleen	122.6 [112.7; 132.5]	16.1 [14.6; 17.7]
	Renal cortex	169.9 [157.0; 186.1]	17.2 [14.9; 20.4]
	Muscle	54.1 [49.6; 60.3]	15.9 [13.8; 17.6]
	Subcutaneous	-91.2 [-96.7; -76.8]	14.5 [12.6; 15.9]
	Air	-999.0 [-999.5; -998.4]	9.2 [8.0; 10.9]
90	Liver metastases	43.8 [31.9; 55.5]	16.0 [14.2; 17.6]
	Liver parenchyma	90.6 [83.2; 100.1]	15.3 [13.8; 17.2]
	VCI	115.3 [102.9; 125.6]	15.1 [13.9; 16.9]
	Aorta	121.3 [112.3; 139.4]	14.3 [12.8; 16.4]
	Portal vein	133.8 [123.9; 157.3]	17.7 [15.8; 20.1]
	Spleen	101.7 [93.9; 111.1]	15.8 [14.3; 17.2]
	Renal cortex	133.0 [122.3; 144.3]	16.1 [14.4; 19.1]
	Muscle	50.9 [46.4; 55.7]	15.5 [13.7; 17.2]
	Subcutaneous	-86.0 [-91.2; -73.2]	13.8 [12.5; 15.5]
	Air	-999.0 [-999.6; -998.3]	9.0 [8.0; 10.7]
100	Liver metastases	41.0 [29.7; 51.8]	15.7 [13.9; 17.3]
	Liver parenchyma	81.7 [74.4; 87.9]	15.2 [13.6; 17.1]
	VCI	94.5 [86.9; 104.4]	14.8 [13.7; 16.9]
	Aorta	101.1 [91.9; 115.0]	14.3 [12.8; 16.1]
	Portal vein	109.8 [100.6; 128.2]	17.2 [15.6; 19.8]
	Spleen	86.2 [80.9; 96.3]	15.6 [14.1; 17.0]
	Renal cortex	107.4 [99.4; 117.3]	15.7 [13.7; 18.3]
	Muscle	49.1 [43.7; 52.1]	15.5 [13.6; 17.1]
	Subcutaneous	-82.5 [-87.7; -70.6]	13.5 [12.2; 15.3]
	Air	-999.1 [-999.7; -998.3]	9.1 [8.0; 10.6]
110	Liver metastases	39.6 [27.6; 48.4]	15.5 [13.8; 17.0]
	Liver parenchyma	74.1 [67.5; 79.2]	15.1 [13.5; 17.0]
	VCI	81.3 [73.6; 89.4]	14.6 [13.5; 16.9]
	Aorta	86.7 [79.5; 98.1]	14.2 [13.0; 16.0]
	Portal vein	92.5 [84.8; 106.8]	17.1 [15.4; 19.7]
	Spleen	75.6 [71.7; 84.2]	15.5 [14.0; 16.9]
	Renal cortex	89.9 [83.0; 98.7]	15.5 [13.4; 18.0]
	Muscle	47.6 [42.1; 50.0]	15.5 [13.5; 16.9]
	Subcutaneous	-80.2 [-85.0; -68.9]	13.4 [12.1; 15.0]
	Air	-999.1 [-999.7; -998.2]	9.1 [8.0; 10.6]
130	Liver metastases	36.3 [25.2; 44.5]	15.5 [13.6; 17.1]

	Liver parenchyma	65.5 [59.4; 70.3]	14.9 [13.5; 16.9]
	VCI	63.2 [56.3; 69.8]	14.6 [13.3; 16.9]
	Aorta	67.8 [61.9; 76.9]	14.3 [12.9; 16.0]
	Portal vein	71.2 [64.0; 82.4]	17.0 [15.3; 19.5]
	Spleen	62.0 [58.8; 68.3]	15.4 [14.0; 17.0]
	Renal cortex	66.9 [60.4; 74.4]	15.4 [13.2; 17.8]
	Muscle	45.6 [40.9; 47.9]	15.5 [13.5; 16.8]
	Subcutaneous	-76.7 [-81.8; -66.8]	13.2 [11.9; 14.8]
	Air	-999.1 [-999.8; -998.2]	9.1 [8.0; 10.6]
150	Liver metastases	34.1 [24.4; 42.2]	15.4 [13.5; 17.0]
	Liver parenchyma	60.0 [54.3; 65.2]	14.9 [13.5; 16.9]
	VCI	52.9 [46.3; 59.8]	14.5 [13.2; 16.8]
	Aorta	56.6 [51.7; 65.4]	14.3 [12.9; 15.9]
	Portal vein	58.7 [51.6; 69.2]	17.0 [15.4; 19.3]
	Spleen	54.8 [51.4; 60.0]	15.5 [14.1; 17.0]
	Renal cortex	53.3 [47.8; 60.7]	15.2 [13.3; 17.7]
	Muscle	43.8 [40.1; 46.8]	15.5 [13.4; 16.9]
	Subcutaneous	-75.0 [-80.0; -65.5]	13.1 [11.9; 14.7]
	Air	-999.1 [-999.9; -998.2]	9.1 [8.0; 10.6]
170	Liver metastases	33.3 [24.1; 40.6]	15.4 [13.5; 16.9]
	Liver parenchyma	56.7 [51.5; 62.0]	14.9 [13.5; 16.9]
	VCI	46.6 [40.5; 52.3]	14.5 [13.2; 16.9]
	Aorta	50.1 [45.0; 57.7]	14.3 [12.8; 16.0]
	Portal vein	50.8 [43.3; 60.5]	17.0 [15.3; 19.2]
	Spleen	50.0 [46.9; 54.9]	15.6 [14.1; 17.0]
	Renal cortex	45.3 [39.3; 51.7]	15.2 [13.3; 17.7]
	Muscle	42.8 [39.2; 46.1]	15.5 [13.4; 16.9]
	Subcutaneous	-74.1 [-78.9; -64.6]	13.1 [11.8; 14.7]
	Air	-999.1 [-999.9; -998.2]	9.1 [8.0; 10.7]
190	Liver metastases	32.7 [23.8; 39.5]	15.3 [13.4; 16.9]
	Liver parenchyma	54.4 [49.0; 60.0]	14.9 [13.4; 16.9]
	VCI	42.2 [37.5; 47.9]	14.4 [13.1; 17.0]
	Aorta	45.7 [41.3; 52.4]	14.3 [12.9; 16.0]
	Portal vein	46.0 [38.1; 54.7]	16.9 [15.3; 19.2]
	Spleen	47.2 [44.0; 51.5]	15.6 [14.1; 17.0]
	Renal cortex	40.0 [34.1; 46.3]	15.2 [13.4; 17.7]
	Muscle	42.4 [38.6; 45.5]	15.5 [13.4; 16.8]
	Subcutaneous	-73.4 [-78.2; -64.1]	13.0 [11.7; 14.7]
	Air	-999.1 [-1000.0; -998.2]	9.1 [8.0; 10.6]
**EID-CT**			
	Liver metastases	46.2 [32.4; 70.1]	19.2 [16.5; 22.1]
	Liver parenchyma	122.1 [108.4; 140.6]	18.2 [15.8; 22.6]
	VCI	169.8 [148.3; 213.1]	18.9 [15.5; 22.6]
	Aorta	172.5 [146.7; 203.9]	14.8 [12.9; 19.1]
	Portal vein	201.4 [173.8; 234.9]	19.6 [16.7; 23.0]
	Spleen	136.8 [123.5; 156.6]	19.4 [16.6; 23.3]
	Renal cortex	195.5 [163.1; 216.1]	20.9 [17.7; 24.5]
	Muscle	52.7 [48.9; 58.4]	19.4 [15.9; 22.4]
	Subcutaneous	-108.2 [-113.7; -86.1]	17.1 [14.6; 20.6]
	Air	-1000.0 [-1000.9; -998.8]	9.5 [7.9; 11.8]

Data shown as median [interquartile range].
