# Supplementary material for: Optimal Conspicuity of Liver Metastases in Virtual Monochromatic Imaging Reconstructions on a Novel Photon-Counting Detector CT—Effect of keV Settings and BMI
Source: Diagnostics (Basel). 2022 May 14;12(5):1231. doi: 10.3390/diagnostics12051231 (PMC9140684; doi:10.3390/diagnostics12051231)
Supplement: Supplementary file 1 [file diagnostics-12-01231-s001.zip › Table S3.pdf]

Supplemental Table S3 Median image noise at different keV levels, divided into two groups (according to BMI)

<table>
<tr><th></th><th colspan="3">PCD-CT</th><th colspan="3">EID-CT</th></tr>
<tr><th>keV</th><th colspan="2">Noise (median SD)</th><th>P-Value</th><th colspan="2">Noise (median SD)</th><th>P-Value</th></tr>
<tr><td></td><td>≤ 23.88 kg/m²</td><td>> 23.88 kg/m²</td><td></td><td>≤ 23.88 kg/m²</td><td>> 23.88 kg/m²</td><td></td></tr>
<tr><td>40</td><td>29.5 (23.7-34.7)</td><td>32.3 (26.8-38.2)</td><td><0.001</td><td rowspan="14">16.6 (13.1-20.3)</td><td rowspan="14">18.6 (14.4-22.8)</td><td rowspan="14"><0.001</td></tr>
<tr><td>45</td><td>26.2 (21.2-30.9)</td><td>28.5 (23.8-33.2)</td><td><0.001</td></tr>
<tr><td>50</td><td>23.4 (19.3-27.3)</td><td>25.7 (21.4-30.0)</td><td><0.001</td></tr>
<tr><td>55</td><td>21.3 (17.6-24.8)</td><td>23.1 (19.3-27.1)</td><td><0.001</td></tr>
<tr><td>60</td><td>19.5 (16.3-22.8)</td><td>21.3 (17.8-24.7)</td><td><0.001</td></tr>
<tr><td>70</td><td>16.2 (13.7-18.7)</td><td>17.2 (14.2-19.7)</td><td>0.003</td></tr>
<tr><td>80</td><td>15.0 (12.9-17.1)</td><td>15.7 (13.4-17.9)</td><td>0.007</td></tr>
<tr><td>90</td><td>14.4 (12.5-16.5)</td><td>15.2 (13.0-17.2)</td><td>0.004</td></tr>
<tr><td>100</td><td>14.1 (12.3-16.3)</td><td>15.0 (12.9-17.0)</td><td>0.002</td></tr>
<tr><td>110</td><td>14.0 (12.2-16.2)</td><td>15.0 (12.8-16.9)</td><td>0.001</td></tr>
<tr><td>130</td><td>13.9 (12.0-16.1)</td><td>14.9 (12.8-16.8)</td><td><0.001</td></tr>
<tr><td>150</td><td>13.9 (11.9-16.1)</td><td>14.9 (12.7-16.8)</td><td><0.001</td></tr>
<tr><td>170</td><td>13.9 (11.9-16.1)</td><td>14.9 (12.7-16.8)</td><td><0.001</td></tr>
<tr><td>190</td><td>13.9 (11.8-16.1)</td><td>14.9 (12.7-16.9)</td><td><0.001</td></tr>
</table>

Median of all SD’s measured in all ROI’s. Data shown as median (interquartile range), P-Value < 0.0033 shown in *Italics*
