# Supplementary material for: Optimal Conspicuity of Liver Metastases in Virtual Monochromatic Imaging Reconstructions on a Novel Photon-Counting Detector CT—Effect of keV Settings and BMI
Source: Diagnostics (Basel). 2022 May 14;12(5):1231. doi: 10.3390/diagnostics12051231 (PMC9140684; doi:10.3390/diagnostics12051231)
Supplement: Supplementary file 1 [file diagnostics-12-01231-s001.zip › Table S4.pdf]

Supplemental Table S4 Spearman Correlations between BMI, image noise, TLR and CNR

		PCD-CT		EID-CT	
	**keV**	**rho**	**P-Value**	**rho**	**P-Value**
**BMI and image noise**	**40**	0.281	0.006	0.252	0.011
	**45**	0.251	0.015		
	**50**	0.232	0.025		
	**55**	0.179	0.084		
	**60**	0.121	0.244		
	**70**	0.016	0.881		
	**80**	0.116	0.264		
	**90**	0.166	0.110		
	**100**	0.207	0.045		
	**110**	0.220	0.033		
	**130**	0.250	0.015		
	**150**	0.263	0.010		
	**170**	0.268	0.009		
	**190**	0.265	0.010		
**BMI and TLR**	**40**	0.083	0.581	0.023	0.876
	**45**	0.082	0.585		
	**50**	0.070	0.641		
	**55**	0.078	0.603		
	**60**	0.101	0.500		
	**70**	0.111	0.456		
	**80**	0.136	0.363		
	**90**	0.132	0.378		
	**100**	0.145	0.330		
	**110**	0.142	0.341		
	**130**	0.131	0.380		
	**150**	0.137	0.358		
	**170**	0.140	0.350		
	**190**	0.134	0.367		
**BMI and CNR**	**40**	-0.232	0.116	-0.399	0.004
	**45**	-0.224	0.131		
	**50**	-0.217	0.142		
	**55**	-0.222	0.133		
	**60**	-0.191	0.197		
	**70**	-0.187	0.208		
	**80**	-0.216	0.145		
	**90**	-0.237	0.108		
	**100**	-0.219	0.139		
	**110**	-0.223	0.131		
	**130**	-0.254	0.085		
	**150**	-0.275	0.061		
	**170**	-0.270	0.066		
	**190**	-0.255	0.084		

Significant P-Value < 0.0033 shown in *Italics.*
