# Supplementary figures and images for: Optimal Conspicuity of Liver Metastases in Virtual Monochromatic Imaging Reconstructions on a Novel Photon-Counting Detector CT—Effect of keV Settings and BMI
Source: Diagnostics (Basel). 2022 May 14;12(5):1231. doi: 10.3390/diagnostics12051231 (PMC9140684; doi:10.3390/diagnostics12051231)

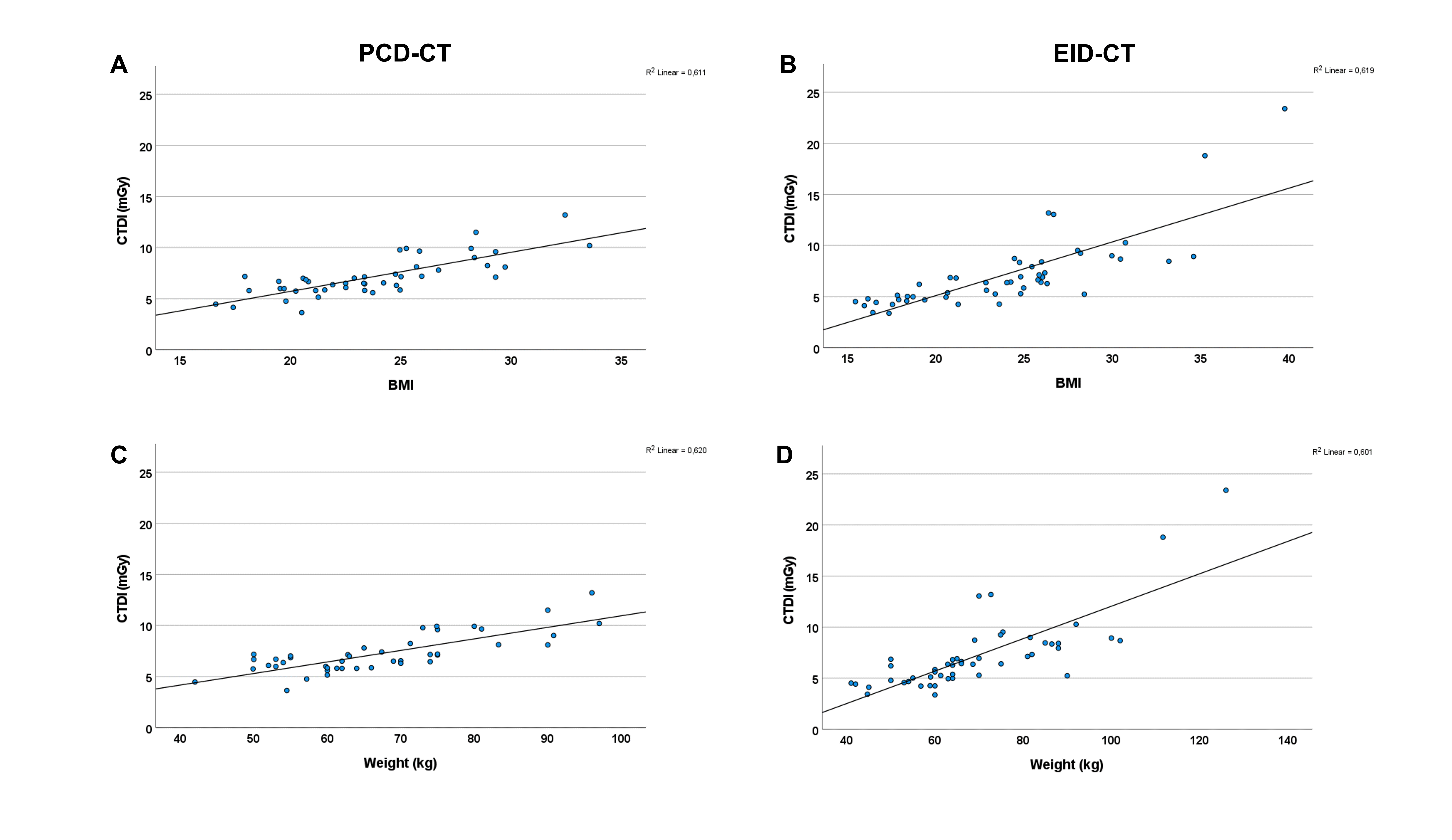

Supplement: Supplementary file 1 [file diagnostics-12-01231-s001.zip › Figure S1.gif]
